# Experiences of healthcare personnel on the efficacy of artemisinin-based combination therapy and malaria diagnosis in hospitals in Uganda

**DOI:** 10.1186/s12936-023-04800-2

**Published:** 2023-11-27

**Authors:** Moses Ocan, Racheal Bakubi, Mordecai Tayebwa, Joan Basemera, Sam Nsobya

**Affiliations:** 1 Department of Pharmacology & Therapeutics, College of Health Sciences, Makerere University, P. O. Box 7072, Kampala, Uganda; 2 Department of Health Policy, Planning and Management, College of Health Sciences, Makerere University, P. O. Box 7072, Kampala, Uganda; 3 Grants office, College of Health Sciences, Makerere University, P. O. Box 7072, Kampala, Uganda; 4 Department of Immunology and Molecular Biology, College of Health Sciences, P. O. Box 7072, Kampala, Uganda; 5 Infectious Disease Research Collaboration (IDRC), P. O. Box 7475, Kampala, Uganda

**Keywords:** Test-and-treat, Malaria, Rapid diagnostic test, Microscopy, Malaria, Policy

## Abstract

**Background:**

The risk of widespread resistance to artemisinin-based combination therapy (ACT) remains high in Uganda following detection of *Plasmodium falciparum* parasites with delayed artemisinin clearance genotype and phenotype. Establishment of context specific interventions to mitigate emergence and spread of artemisinin resistance is thus key in the fight against malaria in the country. The aim of this study was to explore the experiences of healthcare personnel on malaria diagnosis and self-reported efficacy of ACT in the management of malaria symptomatic patients in hospitals in low and high malaria transmission settings in Uganda.

**Methods:**

This was a qualitative study in which data was collected from healthcare personnel in hospitals using key informant interviews. The key informant interview guide was developed, pre-tested prior to use and covered the following areas, (i) sociodemographic characteristics, (ii) malaria diagnosis (clinical and parasite based), (iii) quality-assured artemisinin-based combination therapy, (iv) malaria patient follow-up, (v) artemisinin resistance, (vi) anti-malarial self-medication. Data was entered in Atlas.ti *ver* 9.0 and analysis done following a framework criterion.

**Results:**

A total of 22 respondents were interviewed of which 16 (72.7%) were clinicians. Majority, 81.8% (18/22) of the respondents were male. The following themes were developed from the analysis, malaria diagnosis (procedures and challenges), use of malaria laboratory test results, malaria treatment in hospitals, use of quality assured ACT (QAACT) in malaria treatment, and efficacy of ACT in malaria treatment.

**Conclusion:**

Most healthcare personnel-initiated malaria treatment after a positive laboratory test. Cases of malaria patients who report remaining symptomatic after prior use of ACT exist especially in high malaria transmission settings in Uganda. There is need for regular monitoring of artemisinin resistance emergence and spread in the country.

## Background

Malaria remains one of the leading causes of morbidity and mortality in Uganda with an overall prevalence of about 19% [1]. The disease is endemic in most parts of the country (~ 95%) with an all year-round transmission [2, 3]. Malaria accounts for 30–50% of all out-patient visits in health facilities, 15–20% of all hospital admissions, and up-to 20% of all hospital deaths [1]. Globally, there was an increase of two million malaria cases between 2020 and 2021 an indicator of the challenges faced by the current control and elimination measures [4]. Between 2020 and 2021, Uganda was one of the four countries who contributed nearly half of all malaria cases globally [4].

With the high burden of malaria in Uganda, knowledge of healthcare personnel on effective malaria case management is key for the implementation of national malaria control strategies. The Ministry of Health (MoH) developed the national malaria case management guidelines which require testing of all fevers prior to treatment initiation. Artemether–lumefantrine (AL) and amodiaquine–artesunate (AQ+AS) are the preferred and the alternative first-line agents for the treatment of uncomplicated malaria, respectively. Furthermore, dihydroartemisinin + piperaquine (DP) is the second-line agent for uncomplicated malaria [5]. Previous studies have reported low adherence to the national malaria case management guidelines in the country which could potentially contribute to inappropriate treatment outcomes [6].

The ACT is the mainstay of malaria case management globally [4]. However, in Uganda recent studies have detected evidence of possible emergence of *Plasmodium falciparum* parasites with reduced susceptibility to ACT [7, 8]. The *Pfkelch13* mutation, R539T recently detected in a study by Balikagala et al. [7] in northern Uganda was further confirmed to confer artemisinin resistant phenotype among *P. falciparum* parasites [8]. With no known effective alternative to ACT for malaria case management, emergence and spread of parasite resistance poses a threat to malaria control and eradication efforts in the country.

Healthcare personnel play a key role as first line responders in the fight against malaria. This study explored the experiences of healthcare personnel on patient self-reported efficacy of ACT and malaria diagnosis in hospitals in high and low malaria transmission settings in Uganda.

## Methods

### Study design and setting

This was an exploratory qualitative study conducted in public and private hospitals in low (Kabale and Mbarara districts) and high (Apac and Tororo districts) malaria transmission settings in Uganda. Data was collected from June–December 2021.

### Study population and sampling

The study was done among healthcare personnel (clinicians and laboratory technicians). The sample size estimation was guided by the principle of saturation where the interviews were conducted up to a point where further interviews did not generate new perspectives or information. The hospitals with high malaria patient load in the study districts were purposively selected for inclusion in the study.

### Data collection procedure

In each of the included hospitals, healthcare personnel (clinicians and laboratory technicians) were interviewed. Data collection was done through key informant interviews (KIIs). The KII guide was developed using information from the World Health Organization Global malaria report and national malaria treatment policy. The KII guide collected data on the following areas, (i) socio-demographic characteristics, (ii) malaria diagnosis (criteria and associated challenges), (iii) how malaria test results are used by healthcare personnel in the hospital, (iv) treatment of malaria cases, (v) use of quality-assured artemisinin-based combination therapy (QAACT), (vi) artemisinin resistance (efficacy of ACT). The interview guide was pretested among ten (10) clinicians in Kampala city. The pretest information was used to improve the tool and ensure clarity of the discussion guides. The interviews were conducted by two research assistants, a social scientist (OW) and a clinical pharmacologist (OM) who was the lead researcher. The research assistant was trained on the study protocol and the interview guide prior to field data collection. The interviews were conducted in English and audio recorded using a voice recorder (SONY, ICD-PX470, China) to ensure all data was captured accurately. Additionally, field notes were taken during each of the interviews.

### Data management and analysis

At the end of each data collection day, audio recordings were transcribed verbatim into microsoft word and additional information from field notes included in the transcripts. A total of 10 transcripts were randomly selected by the principal investigator and compared with the audio recordings to check for accuracy and consistency. To facilitate coding and analysis, the transcribed data was entered into Atlas.ti software *ver* 9.0. Each transcript was given a unique participant identification number, a code, and a date to maintain anonymity. The analysis followed a framework criterion [9] which comprised of the following five phases. Phase one, familiarization with the data. In this phase, the interview transcripts were reviewed by the analyst to become acquainted with the data and look for ideas, patterns, and the developing themes. Phase two, development of a coding framework (identifying themes) that is informed by the ideas that emerged from the familiarization phase of the analysis. The code book was developed by two independent team members (RB and MK), and it was reviewed by the principal investigator (OM). Phase three, data coding/indexing. This involved identification, organizing and labelling of data into meaningful groups. The developed codes were reviewed by the principal investigator. Phase four, charting and summarizing of coded data. This involved re-organization of coded data to identify emerging themes. Phase five, interpretation/mapping of data. In this phase, data was interpreted by identifying key concepts separately and then compiled them to identify any relationships that may exist.

## Results

### Characteristics of study respondents

A total of 22 respondents were interviewed of which 16 (72.7%) were clinicians and 6 (27.3%) were laboratory technicians in the hospitals. Majority, 81.8% (18/22) of the study participants were male.

### Themes

The following themes were developed from the analysis,

(i) Procedures and challenges of malaria diagnosis,
(ii) use of malaria laboratory test results,
(iii) malaria treatment in hospitals,
(iv) use of quality-assured ACT (QAACT) in malaria treatment, and.
(v) efficacy of ACT in malaria treatment (resistance).

### Malaria diagnosis (procedures and challenges)

#### Process of malaria diagnosis and treatment initiation in the study hospitals

The process of diagnosing malaria among symptomatic patients did not vary across study hospitals. Healthcare personnel reported assessing and documenting patients’ history upon arrival at the facility. All patients that presented with mild or severe signs and symptoms of malaria are usually tested for malaria using microscopy or malaria rapid diagnostic test (RDT). Study participants reported that malaria treatment would be initiated only if the patient had a positive laboratory test result, either RDT or microscopy. However, participants reported that in a few instances patients with clinical signs and symptoms of malaria but with negative microscopy or RDT results are also initiated on treatment.

*…in this facility, malaria is diagnosed as follows. When a patient comes, we take biodata, after that, we take a proper history, proper physical examination and then we do the investigation and most times the investigation done is about the blood slide, microscopy. It is only at awkward hours like at night when we do RDT. Actually, at the hospital level, we try as much as possible not to do RDT…* ***(KII, Clinician)***.

#### Types of tests used for malaria diagnosis in the study hospitals

National malaria policy requires use of parasitological diagnosis in malaria case management. Study participants reported using microscopy, RDT and clinical symptoms for malaria diagnosis in the study hospitals. However, most of the study hospitals use primarily RDT for malaria diagnosis.

…….*we use RDT, blood slides for microscopy……the other is clinical diagnosis but we normally do not use it a lot for malaria diagnosis. Most clinicians opt for RDT for malaria diagnosis…* ***(KII, Clinician)***.

Microscopy and RDT were the most common tests for malaria diagnosis across all study hospitals. These are usually performed after obtaining patient history. Study participants however reported some instances where only clinical symptoms were used for malaria diagnosis which contradicts the requirement for parasitological test by the national malaria treatment policy. Whereas microscopy is considered the gold standard for malaria diagnosis, in all the study hospitals, RDT was the most common test used for malaria diagnosis.

#### Use of microscopy in malaria diagnosis in the study hospitals

Globally, microscopy is a gold standard test for malaria diagnosis [4]. The participants noted that they preferred microscopy test because of its ability to quantify malaria parasites and thus help clinicians assess the severity of malaria among symptomatic patients. This is helpful as it guides in selecting appropriate malaria treatment. The study participants reported that patients with a history of recent intake of ACT prior to hospital visit, either by self or from another facility, are usually subjected to microscopy for malaria diagnosis. They reported that RDT tests tend to provide negative results for such patients resulting in improper treatment.

*………patients presenting with symptoms of malaria after initial treatment I mean those whose symptoms do not improve after earlier treatment with AL, we normally request for microscopy to be conducted for malaria diagnosis…* ***(KII, clinician)***.

#### Malaria rapid diagnostic test (RDT)

The participants reported using *Plasmodium falciparum histidine rich protein 2 (PfHRP-2)* based rapid test for malaria diagnosis. Majority of the hospitals commonly used RDT for malaria diagnosis and in most cases as a point of care test.

*…In the facility, the common one is the RDT although our standard is the microscopy…For point of care, because we have different sites where we use these RDTs commonly for instance, the casualty ward, the labor suite, there we distribute RDTs and the staffs who are there are well trained, and they can carry out the malaria diagnosis using RDT. But in the main laboratory, we recommend microscopy…* ***(KII, laboratory technician)***.

#### Clinical diagnosis of malaria in the study hospitals

The national guidelines do not recommend use of clinical symptoms for malaria diagnosis. However, some study participants reported use of clinical symptoms for malaria diagnosis. The use of clinical diagnosis was reported by most respondents to be due to, stock out of laboratory supplies and RDT kits as well as the lack of an alternative power supply in cases of power outage. Additionally, when malaria symptomatic patients come to the hospital at night when the laboratory personnel are not present, the healthcare personnel rely on clinical symptoms for malaria diagnosis.

*… we use clinical diagnosis to a minimal level. This is a general hospital, and we are supposed to run in-patient and Out-patient department laboratory services, but you will realize that sometimes the patient comes in the night, and you are a clinician on call and the laboratory person to run the test is not there. So, to a minimal level we are forced to make a clinical diagnosis…* ***(KII, Clinician)***.

#### Challenges of malaria diagnosis in the study hospitals

The respondents described various challenges facing malaria diagnosis in the study hospitals. These included the following.

i) Quality of the RDT test kits: study participants reported cases where the RDT kits supplied were giving negative results for malaria symptomatic patients. However, positive results were encountered whenever the test was redone using microscopy. Further still, it was noted that the RDTs used in hospitals in the country were specific to *P. falciparum* only. Other malaria parasites like *Plasmodium ovale*, *Plasmodium vivax* and *Plasmodium malariae* cannot be detected by the current RDT kits (*PfHRP-2*) used in the hospitals.
  *… I had said most of these RDTs are only specific to P. falciparum. So other species like ovale, vivax and malariae may not show up in the RDT test results. That is one reason. Then, there are some instances where these RDTs that were brought would give you a negative result… So, when you do a quality control check with microscopy, you would find the malaria parasites are there…* ***(KII, Clinician)***.
ii) Human resource gaps: participants across the study hospitals cited gaps with the laboratory human resource. Most of the hospitals reported that they do not have enough qualified staff for proper functioning of the laboratory. Important to note is that the inadequate laboratory staff were reported in all study hospitals. The study participants reported lack of microscopists in the hospitals which affected the conduct and quality of malaria microscopy tests done in the hospitals. Microscopy test require more time to generate results which is a challenge due to gaps in human resource and infrastructure in most hospitals in the country.
iii) Knowledge gaps especially in the use of microscopy: this was emphasized because negative results that are sometimes produced from the laboratory are due to oversight or mistakes when it comes to detecting and quantifying the parasites. Coordination gaps between the laboratory personnel and the clinicians was cited as a key challenge in some facilities. Healthcare personnel from high volume facilities noted that laboratory personnel tend to ignore request for microscopy test by the clinicians and instead provide RDT results or forge microscopy results because of too much workload.
  *…Laboratory entomology assistant needs more training in using the microscope for malaria diagnosis…* ***(KII, Laboratory in-charge)***.
iv) Inadequate infrastructure: respondents noted that the laboratories do not have adequate space and equipment compared to the population that they serve especially in high malaria transmission settings. The laboratory space available is used to perform various tests. In addition, the unstable power supply and lack of alternative power sources affected laboratories in most of the study hospitals. Due to the lack of reliable power supply, the respondents resort to using RDT for malaria diagnosis in the study hospitals. In most of the study hospitals, participants reported either lack of or presence of only non-functioning microscopes.
v) Limited consumables and stock outs of laboratory supplies: hospitals reported stock out of laboratory supplies like reagents that are necessary for running malaria laboratory tests. Stock outs and lack of laboratory supplies was commonly reported by respondents from Apac and Tororo general hospitals (high malaria transmission settings). In the study hospitals, malaria slides are processed using Field stain A and B. However, in most hospitals there was uncoordinated delivery of these stains with only one stain being available in most cases in the study hospitals. In malaria microscopy Field stain A and B are used together for slide processing. This therefore prompted the laboratory technicians to resort to using malaria rapid test for malaria diagnosis (RDT).
  *…Yes, the challenges are there. As I said, one of them is stock outs. We used to do Giemsa, which was so good and very sensitive, but we do not get it from National Medical Stores (NMS), so we rely on these other people who are doing their research. So since last year we didn’t have any active participants in research and so Giemsa was out of stock and then we turned to Field Stain…* ***(KII, Laboratory in-charge)***.
vi) Unreliable power supply in the study hospitals: electricity is needed for the conduct of malaria microscopy. In most hospitals however, unstable supply of electricity was common and was a major barrier to the conduct of malaria microscopy. Respondents noted that hospitals do not have alternative sources of power and, therefore, cannot ensure uninterrupted conduct of malaria microscopy. In such cases, RDT was often relied upon as an alternative to the microscopy.

#### Choice of the malaria diagnostic method among study participants in the hospitals

Participants reported various reasons for their choice of the diagnostic method used for diagnosis of among symptomatic patients. Microscopy tests are used by hospitals as it is a recommended test for malaria in the country. According to the Uganda national malaria control policy, microscopy is the gold standard or reference test for malaria diagnosis. In addition, study respondents noted that the microscopy test is also preferred because of its ability to quantify the number of parasites and the severity of the malaria in patients which helps clinicians to properly treat and manage malaria patients. Further still, respondents noted that for patients that had a history of prior recent medication, either by self or by any other facility, are usually subjected to the microscopy and not RDT. They reported that RDT tests tend to provide negative results for such patients resulting in improper treatment.

*….We are accredited for malaria microscopy. We do microscopy as our gold standard. RDT just comes in as a backup line like maybe when power is gone because, one of the challenges we have in this lab is power back up. We have no power backup. So, when power goes off, we switch on to RDTs….* ***(KII, Laboratory technician)***.

#### Use of laboratory test results and malaria treatment in study hospitals

The study participants reported using laboratory results to help establish the malaria diagnosis and determination of treatment initiation.

*….So, if the lab results have shown that the person has malaria then we go ahead and initiate treatment. And we do not treat if there is no positive laboratory result for malaria…* ***(KII, Clinician)***.

The clinicians reported using laboratory results to determine the kind of antimalarial treatment to prescribe for the patients. For example, patients whose microscopy results showed few malaria parasites (non-severe or uncomplicated malaria) were in most cases initiated on artemether-lumefantrine (AL). While patients who had high parasite load (severe malaria) were initiated (prescribed) on either intravenous artesunate, quinine or intramuscular artemether and, thereafter, an additional 3-day treatment using oral AL.

*…. In this facility, we commonly encounter both uncomplicated and complicated malaria (severe). For uncomplicated malaria, we give tablets of AL or Duocotecxin (Dihydroartemisinin-Piperaquine). For severe malaria depending on the parasites present with other things, maybe parasites present with anemia, we use artesunate as the first line ….* ***(KII, Clinician)***

Participants reported the use of sulfadoxine-pyrimethamine (Fansidar, SP) for malaria prophylaxis in children, pregnant mothers, and sickle cell patients. During antenatal care visits, mothers receive SP for intermittent preventive treatment in pregnancy (IPTp). In case of a malaria diagnosis among pregnant women, quinine and AL were commonly used for treatment.

Clinicians reported using laboratory results in monitoring malaria patients admitted in the hospital. For monitoring malaria treatment, microscopy is done after 24 h following administration of intravenous artesunate. Any reduction in the number of parasites as compared to the initial test is taken as an indicator of positive response to the treatment. Malaria microscopy was conducted among malaria in-patients prior to being discharged from the hospital.

#### Follow up of patients on malaria treatment in the study hospitals

Most clinicians did not follow up patients after 3 days of ACT for malaria. Patients follow up after 3 days of ACT was reported to be a challenge across all study hospitals. Respondents noted that they are not aware of the need to follow up patients treated for malaria using ACT in the hospital. The participants reported the following concerns regarding patient follow-up, (i) distance between the hospital and the patient homes, (ii) Lack of funds, (iii) lack of awareness of the need to follow-up patients after 3 days of ACT use for malaria treatment, (iv) high patient numbers/load.

*….I have not been doing patient follow-up after treatment…I do not even have the means for following up these patients. Airtime, transport, and not everyone has phones…* ***(KII, Clinician)***.

***….****No, we do not request them to comeback after treatment for follow-up, but we health educate them on how to prevent malaria….* ***(KII, Clinician)***.

#### Clinician experiences on self-reported efficacy of ACT by malaria patients seeking treatment in hospitals

According to the healthcare personnel, ACT has still a high efficacy in malaria treatment; self-reported use of other anti-malarial drugs like quinine was also encountered by the healthcare personnel. These were also reported by patients to still be effective medicines for malaria treatment from their experience in the communities.

In addition, participants noted that there has not been much resistance to ACT used in malaria treatment. Study participants reported overuse of IV artesunate by clinicians for malaria treatment as nearly all cases of malaria are being managed using artesunate. This is because of the perception on its efficacy and rapid action. However, study participants reported high prevalence of misuse of anti-malarial agents in the community. More still, some participants reported an increase in the number of malaria cases even after indoor residual spraying (IRS). In a few cases healthcare personnel in study hospitals encountered malaria patients who reported persistence of malaria symptoms even after taking ACT before coming to the hospital.

*…I have not seen so much resistance because most times whenever we give them Artemether-Lumefantrine, they get better…. just in a few instances like two when someone took treatment…came back and they were like worse than how they were before the treatment….* ***(KII, Clinician)***

*….Certainly, because with some of these patients we interact with, by the time they report to you at the hospital, they tell you that they have had some treatment. They’ll at least tell you whether they have had one or two doses of Coartem (ACT) and then you will wonder where they got it from and then they will tell you that it remained from the last time so and so was given….* ***(KII, Clinician).***

#### Awareness of quality-assured artemisinin-based combination therapy (QAACT)

The study found that participants were not aware of the existence of quality assured artemisinin-based combination therapy (QAACT) in the country (‘Green leaf ACT’). The definition of ‘Green leaf ACT’ ranged from drugs given by government facilities to medicines having a picture of green leaf on the container. The participants reported that they did not consider quality of the ACT that they used for malaria treatment since the medicines are supplied by government.

*…me I do not think of quality of the antimalarial medicines because this being a government facility I think government should be able to ensure quality of the medicines that are supplied to the hospitals…* ***(KII, Clinician)***.

#### Self-medication with anti-malarial agents among patients prior to hospital visit

Study participants reported high prevalence of self-treatment among malaria symptomatic patients prior to seeking treatment in the hospitals. Additionally, most of the malaria patients do not complete the dose of the anti-malarial agents. The study findings indicate that chloroquine is being accessed over the counter and used for malaria treatment by most malaria patients in the study districts.

*…There must be a misuse of AL (Coartem) somewhere because people come with a mind that I have used this drug or the child had a fever at night and they took some Coartem and by the time the child comes here, they have already taken Coartem. Self-prescription is a very big problem in this country…* ***(KII, Clinician)***.

***…****Most times these patients when they have fever, they think it is malaria, so they go to the private pharmacy and buy AL…. not even a complete dose, so they come to the hospital after they have self-medicated….* ***(KII, Laboratory technician)***.

Study respondents also reported instances where malaria symptomatic patients first used herbal medicines in managing malaria symptoms prior to coming to hospital. This potentially delays access to effective malaria treatment which could lead to worsening of the disease.

*…Some people they delay managing malaria at home using herbal medicine….and only come to hospital when the symptoms persist which is usually late and malaria has become severe….* ***(KII, laboratory technician)***.

## Discussion

In this study, the healthcare personnel encountered cases where patients reported to hospitals with malaria symptoms following initial prior treatment at home using ACT. Additionally, in a few cases healthcare personnel reported cases where patients reported back to hospital with malaria symptoms that persisted following ACT treatment initiated from the hospital. These were mainly reported in high malaria transmission settings of Tororo and Apac districts. The persistence of symptoms despite ACT use is a pointer to the potential reduction in the efficacy of artemisinin-based combinations in the community. This is like the findings of a previous study by Balikagala et al. [7] in northern Uganda, which also reported indicators of potential emergence of artemisinin resistance in northern Uganda. Although the instances of potential reduction in efficacy of artemisinin-based combinations in malaria treatment remain low in Uganda, this is a potential cause for concern due to lack of alternatives to artemisinin-based agents, the current cornerstone in malaria treatment. This tcalls for regular surveillance to monitor the efficacy of ACT in malaria treatment in the country.

However, in this study no follow-up of patients after 3-days of artemisinin-based combination treatment to monitor the efficacy of ACT with healthcare personnel reporting lack of awareness and resources to enable follow-up of patients on malaria treatment. Following-up malaria patients on ACT is an important component of the artemisinin resistance surveillance efforts. This is especially critical in Uganda with the current reported possible emergence of artemisinin resistance of *P. falciparum* [7, 10]. The risk of artemisinin resistance was further confirmed with reports from some of the study participants where malaria patients on ACT continued being malaria symptomatic even after initial treatment. Inadequate funding, lack of awareness, long distances to patients’ residences, and lack of means of communication were reported by study participants as common barriers to patient follow-up after 3 days of treatment with artemisinin-based combinations. This is similar to findings from previous studies that have reported challenges in implementation of health interventions especially in low-and-middle income countries [11].

The World Health Organization (WHO) recommends parasite-based diagnosis with microscopy or rapid Diagnostic Test (RDT) prior to treatment [4]. In this study the majority of participants reported using malaria laboratory test results as a basis for initiation of treatment. This finding is in line with the recent global malaria report of the WHO which reported increase in utilization of parasite-based diagnosis of malaria globally [4]. A study by Mpimbaza et al. [12] in Busoga, a region with high malaria transmission in Uganda found high level of adherence to the test-and-treat a key component of the national malaria treatment policy. The increase in the practice of test-and-treat in management of malaria symptomatic patients potentially reduces the risk of inappropriate treatment. This is key in the fight against widespread emergence of artemisinin resistance in the country.

Anti-malarial self-medication among malaria symptomatic patients seeking treatment in hospitals was commonly encountered by the healthcare personnel. This is like reports from other studies that have found high prevalence of self-medication using anti-malarial agents in malaria affected regions [13, 14]. Despite being prescription-only medicines, anti-malarial agents are accessed over the counter especially in most malaria affected countries. Self-treatment is likely to lead to delay in accessing appropriate treatment thus worsening of the disease, which potentially leads to wasting of more resources and increased risk of morbidity and mortality [13]. The inability of healthcare personnel to confirm prior use of anti-malarial agents among malaria symptomatic patients seeking treatment in hospitals further affects monitoring of self-medication in the country. This is due to the inadequate laboratory infrastructure a common challenge in most low- and middle-income countries [15].

Following the end of Affordable medicines facility-malaria (AMF-m) programme in 2011, six countries (Nigeria, Ghana, Uganda, Kenya, Tanzania, and Madagascar) adopted a copayment mechanism to help in ensuring continued access to quality-assured artemisinin-based combinations for treatment of malaria especially in the private sector [16]. The artemisinin-based combination therapy (ACT) supplied under the copayment mechanism have a mark of a ‘Green leaf’ on the primary package [16]. However, in this study nearly all healthcare personnel from the study hospitals were not aware of the existence of Quality Assured Artemisinin-based Combination Therapy (QAACT) (‘Green leaf’ ACT) in the country. Lack of awareness of a national programme of copayment mechanism could indicate challenges associated with implementation of the programme especially due to competing priorities coupled with limited funds, a common problem in most low-and middle-income countries [11]. Behaviour change communication (BCC) and training are key components of the implementation of the copayment mechanism, in this study the lack of awareness of the copayment mechanism among the healthcare personnel is likely due to lack of regular training and communication [17].

The study had some limitations, the interviews having been conducted in health facilities the healthcare personnel could have only selected their responses to the discussion points in the interview guide based on what would be expected of them as professionals in the hospitals (social desirability bias). However, the use of probes in the interview guide helped reduce the effects of social desirability bias on the findings of this study.

## Conclusion

Most healthcare personnel treated malaria patients following parasite-based diagnosis in the study hospitals. Malaria patients who remain symptomatic and having *Plasmodium falciparum* parasites despite initial use of ACT for malaria treatment exist especially in high malaria transmission settings in Uganda. There is need for regular surveillance to monitor the efficacy of ACT in malaria management in the country.

## Data Availability

The datasets used and/or analysed during the current study are available from the corresponding author on reasonable request.

## Abbreviations

ACTs: Artemisinin-based combination therapies
AL: Artemether–lumefantrine
K13: Kelch13 propeller gene
QAACT: Quality assured artemisinin combination therapies
AMFm: Afordable medicines facility-malaria
UNCST: Uganda National Council of Science and Technology
WHO: World Health Organization
LMICs: Low-and middle-income countries
RDT: Rapid diagnostic test
